# Global Health Security Alliance (GloHSA)

**DOI:** 10.1017/dmp.2020.70

**Published:** 2020-04-03

**Authors:** John M. Quinn, James M. Wilson, Tracey McNamara, Stefan Goebbels, Jan-Cedric Hansen, Anja Opitz

**Affiliations:** The Global Health Security Alliance (GloHSA) Core Team: Charles University, First Faculty of Medicine, Institute of Hygiene and Epidemiology, Prague Center for Global Health, Prague, Czech Republic; CEO and Founder of M2 Medical Intelligence, Inc., Reno, Nevada; Western University of Health Sciences, College of Veterinary Medicine, Pomona, California; German Armed Forces, affiliated to the Medical Services Division, United Nations Secretariat New York, New York; StratAdviser Ltd, Cindynician Expert-Auditor, London, England; Head of Section International Relations and Security Policy, APB Tutzing, Germany

**Keywords:** health security, European Union, civil-military intervention, COVID-19

In recent days, Europe has become the epicenter of coronavirus disease 2019 (COVID-19). Soaring case fatality rates across European states, disparate public health and global health security response across borders, baseline health-care infrastructure differences, and significant social, economic, and political influences on key decision-making all exacerbate the challenges of this acute crisis. As Europe moves into acute disaster response mode, a unified, oriented, and evidence-based crisis command must be established that goes beyond the established border measures taken and the European Union (EU) export scheme for protective equipment. We propose in this letter the EU mechanism for crisis managed response cycle be initiated immediately to mitigate preventable morbidity and mortality from coronavirus disease 2019 (COVID-19), which includes the North Atlantic Treaty Organization (NATO) and military alliance involvement.

Complete and comprehensive peer reviewed data related to morbidity and mortality for COVID-19 may not be available for many months and likely years. However, public health measures to flatten the curve of case fatality rates differ widely across the affected states and communities. In a maximal effort to defend civilian populations, communities, and regions, self-isolation, social distancing, multiple versions of quarantine, and even full lock downs have been instituted or considered in varying forms; some states may institute Martial Law. Social, economic, and political infrastructure are greatly tested across the EU, and health security once again demonstrates that disease observes no borders or passport color. Indeed, economic stress will come to a breaking point and confidence in European public health and democratic institutions will be greatly challenged; especially as we observe different responses, by individual countries, in the same union against the same coronavirus threat.

The EU Global Strategy points out that the EU is more and more facing hybrid forms of threats, and COVID19 tops the list today. However, the EU struggles to have a unified and integrated civil-military approach to public health crisis, disaster, and disease pandemic that is desperately needed immediately. The European Centre for Disease Prevention and Control (ECDC), an independent agency of the EU whose mission is to strengthen Europe’s defenses against infectious diseases, is closely monitoring the pandemic, providing risk assessments, public health guidance, and advice on response activities to EU Member States and the EU Commission. Within the domain of Civilian-Military interoperability, NATO, with locations throughout Europe, has multiple structures responding separately. The Force Health Protection Branch of the NATO Military Medical Center for Excellence (MILMED COE) is closely monitoring the developments. There are many NATO resources that can additionally be tapped to support the response. Health security intelligence, information sharing, and leadership with command decisions for the EU are completely absent.

Luckily, the EU has an application for that. The Council, or when an EU member state triggers the solidarity clause, can activate the EU Integrated Political Crisis Response Mechanism (IPCR); also referred to as the Crisis Platform, EU Situation Room, Crisis Management Board. This mechanism plays a central role in ensuring both swift and effective mobilization of actors and instruments across the entire EU system, as well as coherence of policies and actions throughout the various phases of the crisis life cycle. The Croatian presidency activated the IPCR in information sharing mode in January 2020 and triggered full activation mode on March 2, 2020.

However, in triggering this mechanism to its full extent, military and civilian resources, including the EU civil protection mechanism, will be liberated and under full command and direction from the Council and the European External Action Service (EEAS); multiple committees and commissioners and military staff would be forced to the table to respond in a unified voice. Full activation mode includes a united and clear structure for response and decision-making, de facto solidarity across the EU for crisis response. Beyond the integrated political crisis response mechanism as it is designed today, the EU, currently still including the United Kingdom, urgently requires a practical tool to analyze and fully apprehend the nexus between the different civilian and military (security and logistic) components of a crisis and disaster, as well as the determinant of the health/wealth concept that bound health systems to the political and economic dimensions of the EU.

As more data become available about case vitality rates, transmissibility, and overall natural history of disease for COVID19, the requirement to maximize information sharing on genomic, clinical, and outcomes will become more apparent. Triggering this crisis mechanism may also lead to further information sharing across platforms, public health infrastructure, socialized medical systems, and integrated with defense health structures. The time for action is now. The time for open and unified policy of how best to mitigate disease spread is required now. In order to provide economic, social, and political unity and confidence in democratic institutions of the EU, this must be done now.

Expansive coordination is essential for the EU, for the sake of both the individual nation states and the collective community. The model is applicable, however, beyond the boundaries of the EU. COVID19 is a global challenge, which mandates a global response. The precise coordinative mechanisms may vary, depending on national law and tradition, but the need for unity of effort has never been greater. Failure in this regard would exact a price, measured by the cost of millions of lives.

